# The Effects of Throat Packing and Gender on Postoperative Nausea, Vomiting, and Sore Throat in Septorhinoplasty Patients: A Randomized Controlled Trial

**DOI:** 10.1007/s00266-026-05750-1

**Published:** 2026-03-23

**Authors:** Tankut Uzun, Mehmet Ekrem Zorlu, Berrak Karatan, Togay Müderris

**Affiliations:** 1 Department of Otolaryngology and Head & Neck Surgery, Faculty of Medicine, Cigli Training and Research Hospital, Izmir Bakircay University, Izmir, Turkey; 2 Department of Otolaryngology and Head & Neck Surgery, Faculty of Medicine, Gazi University, Ankara, Turkey; 3 Department of Plastic Reconstructive and Aesthetic Surgery, Istanbul University Cerrahpasa Faculty of Medicine, Istanbul, Turkey

**Keywords:** PONV, Sore throat, Pharyngeal pack, Septorhinoplasty, Gender

## Abstract

**Background:**

In satisfaction-oriented procedures such as septorhinoplasty, postoperative nausea and vomiting (PONV) and sore throat may adversely affect the hospital discharge process and delay the patient’s return to daily life. To date, there is no conclusive evidence demonstrating the positive impact of throat pack placement on these postoperative symptoms in septorhinoplasty patients, and the role of gender in these outcomes remains insufficiently explored.

**Methods:**

PONV and sore throat were investigated in a randomized, prospective, double-blind study involving 152 patients undergoing elective septorhinoplasty, who were divided into two groups based on the presence or absence of throat packing. Additionally, the effect of gender on these postoperative complaints was also investigated.

**Results:**

Throat packing did not reduce PONV; however, it was associated with an increase in postoperative sore throat during the early postoperative period. Additionally, both PONV and postoperative sore throat were observed to be more common in female patients.

**Conclusion:**

In our study, consistent with previous research, throat packing was not found to reduce PONV and was associated with an increased incidence of postoperative throat pain. Therefore, throat packing is not routinely indicated in elective septorhinoplasty. Additionally, given that PONV and throat pain were more frequently observed in female patients, we recommend the early adjustment of postoperative medication protocols to address these symptoms more effectively in this patient group.

- Throat pack placement in septorhinoplasty did not reduce PONV.
- Throat pack use was associated with a trend toward increased early postoperative sore throat.
- Female patients consistently reported higher rates of PONV and sore throat than males.
- To our knowledge, this is the first randomized controlled trial to specifically evaluate the influence of gender on PONV and postoperative sore throat in septorhinoplasty patients.

**Level of Evidence I:**

This journal requires that authors assign a level of evidence to each article. For a full description of these Evidence-Based Medicine ratings, please refer to the Table of Contents or the online Instructions to Authors  www.springer.com/00266.

## Introduction

Postoperative nausea and vomiting (PONV) is common significant complication following surgical procedures, particularly nasal surgeries [1, 2]. Although PONV is a multifactorial condition, intraoperative bleeding is a significant factor that contributes to its occurrence [3]. Due to the rich vascularization of the nasal region, significant bleeding can occur during nasal surgeries, and endotracheal tubes may not fully prevent blood aspiration [4, 5]. Pharyngeal tampons may be an effective option for reducing PONV by preventing blood aspiration; however, concerns regarding postoperative sore throat have limited their widespread use [3]. In septorhinoplasty patients, postoperative PONV and sore throat can impact hospital stay duration and delay the patient’s return to daily activities following surgery. There is no consensus on the use of pharyngeal packs in septorhinoplasty, a widely performed procedure that prioritizes patient satisfaction. Unfortunately, the necessity and effectiveness of throat pack placement have not been sufficiently emphasized in the studies conducted on this subject.

Numerous studies across various surgical procedures have demonstrated that PONV and postoperative sore throat are more prevalent in women [6–8]. The higher incidence of PONV in female patients has been attributed to the effects of estrogen and progesterone on serotonergic and dopaminergic pathways in the central nervous system, which lower the threshold for nausea and vomiting, as well as to sex-related differences in the pharmacokinetics of antiemetic agents and psychosocial factors [6, 7]. In addition, it is widely recognized that women’s narrower tracheal anatomy, the mucosal permeability-enhancing effects of estrogen, differences in pain perception, and psychological factors contribute to the increased incidence of postoperative sore throat [8, 9].

Despite these observations, no comprehensive study has specifically investigated the influence of gender on PONV and sore throat in septorhinoplasty, a satisfaction-oriented procedure.

In this study, we aimed to objectively evaluate the effects of throat packing, age, and gender on PONV and sore throat. Based on our findings, we believe that individualizing treatment strategies according to patient-specific factors can enhance patient satisfaction and facilitate a quicker return to daily life following septorhinoplasty surgery.

## Methods

Our study was conducted with the approval of the Izmir Bakircay University Clinical Research Ethics Committee (25/10/2023-1269) and in accordance with the Helsinki Declaration. Informed consent was obtained from all patients participating in the study.

The study included patients aged 18–60 years, classified as ASA 1–2, with a BMI <30 kg/m^2^, who underwent septorhinoplasty in our clinic between November 2023 and November 2024. Patients with chronic conditions other than ASA 1-2 (such as diabetes mellitus, heart failure, or liver/kidney disease), a personal or family history of malignant hyperthermia, neuromuscular disorders, opioid, alcohol, or other substance use disorders, as well as women with active menstruation on the day of surgery or breastfeeding women, individuals with known drug allergies, those on anticoagulant therapy, non-smokers, and patients with a history of motion sickness or previous episodes of nausea and vomiting were excluded from the study. Non-smokers were excluded from the study, as smoking has been reported to reduce the incidence of postoperative nausea and vomiting [7]. In addition to excluding patients with a prior history of nausea and vomiting, we screened for symptoms suggestive of gastroesophageal reflux or other gastric pathology during preoperative history-taking (e.g., nausea, epigastric discomfort, heartburn/burning sensation, and bitter taste/regurgitation). Patients reporting clinically significant reflux-related symptoms were not included, to minimize potential confounding in nausea-related outcomes.

Patients were also excluded if they had experienced difficult intubation due to the risk of throat trauma, required multiple intubation attempts, or did not meet the inclusion criteria. In the study, power analysis determined that a minimum of 50 patients per group was required to evaluate PONV with a statistical power of 90%.

**Fig. 1:**
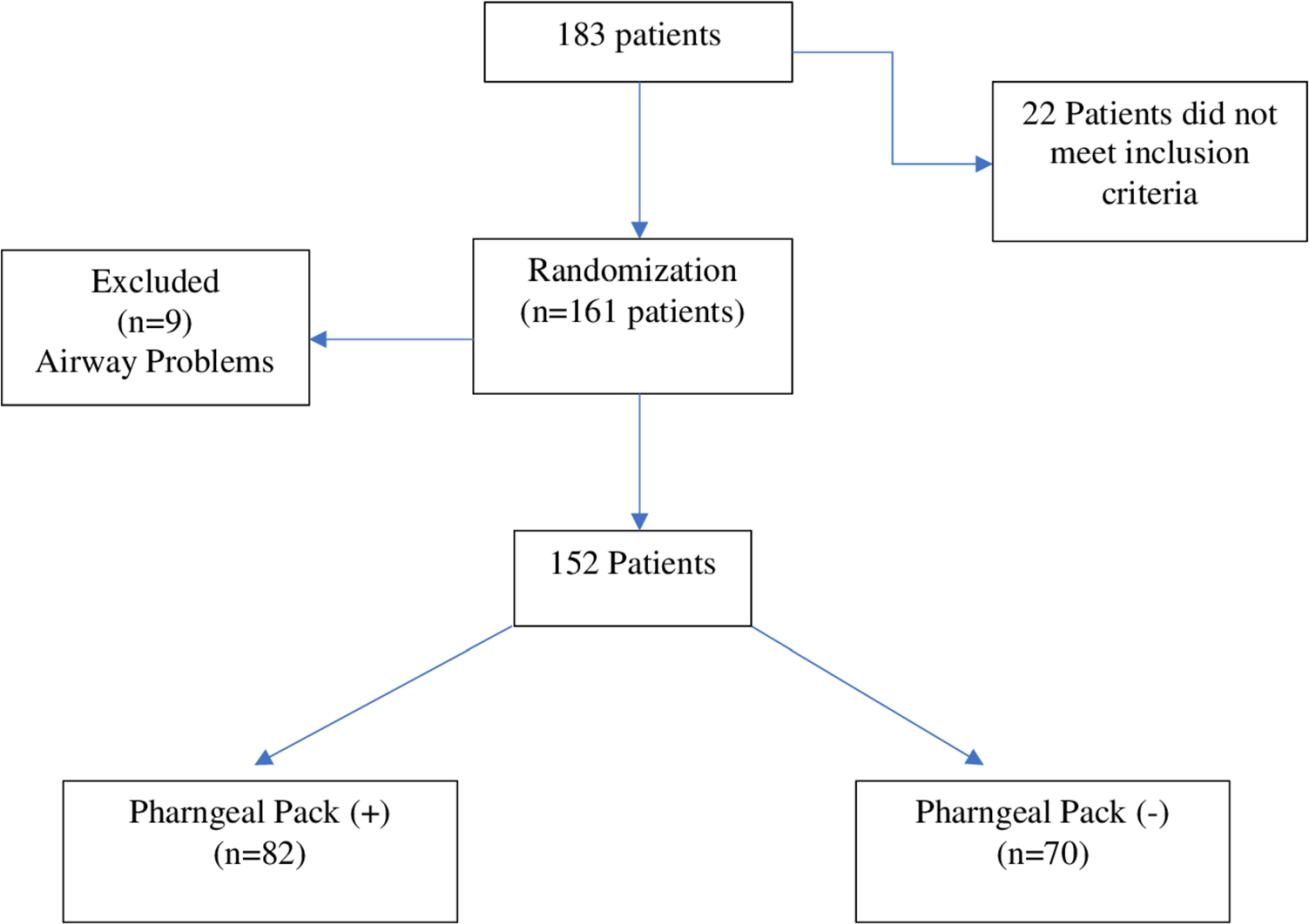
Patient distribution of the study

Among the 183 patients who underwent septorhinoplasty within the study period, 161 patients met the inclusion criteria and were enrolled in the study. Nine patients were excluded from the study due to airway complications. The study was completed with 152 out of the initial 183 patients, based on the application of the inclusion and exclusion criteria (Fig. 1). Patients included in the study were randomly assigned to groups using computer-generated randomization. Randomization was performed using a computer-generated allocation list concealed in sequentially numbered, sealed, opaque envelopes, opened only after patient enrollment. No post-randomization dropouts occurred, and complete data were available for all 152 participants.

Through this randomization process, patients were divided into two groups: those who received throat packing and those who did not. Patients were also grouped by gender to evaluate the potential influence of sex-related factors on postoperative outcomes.

To reduce bias and variance, all patients in both groups underwent a standardized anesthetic and antiemetic approach. Following patient monitoring and the establishment of vascular access, 0.05mg/kg of midazolam was initially delivered. Anesthesia induction was conducted with 2 mg.kg^−1^ intravenous propofol, 1 mcg.kg^−1^ intravenous fentanyl, and 0.6 mg.kg^−1^ intravenous rocuronium. All patients received anesthesia consisting of 1-2% sevoflurane and 50 mcg/h remifentanil in a 50% oxygen–air combination administered by orotracheal intubation. All patients received 8 mg of dexamethasone at the beginning of the operation, and 4 mg of ondansetron along with 1 g of paracetamol was administered 30 minutes before the end of the procedure. Orogastric suctioning was performed in all patients prior to extubation. The trial was designed to assess whether throat packing offers added benefit for PONV prevention on top of standard antiemetic prophylaxis.

The mechanical ventilator settings were modified to a tidal volume of 6–7 mL.kg^−1^ and a PEEP of 2-3 cmH_2_O in volume-control mode, targeting an end-tidal pCO_2_ of 30-35 mmHg. Upon a 20% elevation of the mean arterial pressure above the preoperative baseline, 25 mcg of fentanyl was delivered.

Following intubation, each patient was positioned using a headlight, and a sterile, rolled, moist gauze was carefully inserted with the aid of a curved ovarian clamp and a tongue depressor. Particular attention was given to placing the gauze toward the base of the tongue and the larynx, ensuring no contact with the soft palate or uvula. Gauze of identical size was applied to all patients using a standardized technique. Prior to extubation, the gauze was gently removed under headlight illumination using a curved ovarian clamp and a tongue depressor, with maximum care taken to avoid trauma to the surrounding tissues. Due to the nature of the intervention, anesthesiologists could not be blinded. To reduce potential performance bias, intraoperative management followed a standardized protocol applied to both groups, and opioid administration was titrated according to predefined hemodynamic criteria. Postoperative outcome assessors were blinded to group allocation.

This study was designed as a prospective randomized controlled trial. After being transferred to the ENT ward, patients were assessed by otolaryngology residents who, without knowledge of whether a pharyngeal pack had been placed, applied the PONV impact scale [10] at 6 and 24 hours postoperatively. Additionally, the Visual Analog Scale (VAS, 0–100) was used to evaluate sore throat and nausea.

## Statistical Analysis

For the statistical analysis, descriptive statistics—including mean, standard deviation, median, frequency, percentage, minimum, maximum, and first (Q1) and third (Q3) quartiles—were performed using the IBM SPSS Statistics version 25.0 software (IBM Corp., Armonk, NY, USA). The normality of the distribution was assessed using the Shapiro-Wilk test and visually (histogram, coefficient of variation, skewness/kurtosis, and trendless plots). The independent t test was used to compare normally distributed quantitative variables between groups. The Mann–Whitney U test was used to compare non-normally distributed data between two independent groups. Qualitative variables were compared using Pearson’s chi-square test or Fisher’s exact test, depending on the distribution and expected cell counts. Since multiple comparisons were performed (e.g., across different time points and subgroups), we additionally applied Bonferroni correction to reduce the risk of type I error. Following this adjustment, the significance threshold was set at *p*<0.025 when two time points of the same outcome were compared. A two-tailed p value below these thresholds was considered statistically significant.

## Results

Demographic characteristics of the patients included in the study—such as age, gender, duration of anesthesia, duration of surgery, and mean arterial pressure—are summarized in Tables 1 and 2, categorized by throat packing status and gender, respectively. No statistically significant differences were found between the groups when the specified parameters were compared, indicating that the study was conducted with clinically and demographically comparable groups.

**Table 1:** Demographic data of patients with and without tampons

	Pharyngeal Tampon − (*n*=70)	Pharyngeal Tampon + (*n*=82)	*P*
Gender (male/female)	27/43	27/55	0.47
Age	27.88±9.15	31.26±10.96	0.46
Duration of anesthesia, min	225.57±18,14	216.25±22.76	0.12
Duration of surgery, min	201.14±22.14	192.75±25.76	0.06
Mean arterial pressure, mm Hg	64.85±2.26	62.87±3.75	0.24

Mean±SD

**Table 2:** Demographic data of male and female patients

	Male (*n*=54)	Female (*n*=98)	*P*
Pharyngeal tampon (−/+)	27/27	43/55	0.46
Age	28.11±10.61	30.57±10.03	0.15
Duration of anesthesia, min	222±12.08	219±11.66	0.67
Duration of surgery, min	197.85±8.02	195.62±9.75	0.64
Mean arterial pressure, mm Hg	66.14±2.67	61.75±2.43	0.23

Mean±SD

Among the 152 patients included in the study, 54 (35.5%) were male, and 98 (64.5%) were female. Pharyngeal tampons were not placed in 70 patients (46.1%), whereas 82 patients (53.9%) received tampon placement. The age range of the patients included in the study was 18 to 58 years. Mean arterial pressure values ranged from a minimum of 58 mmHg to a maximum of 70 mmHg. The minimum durations of anesthesia and surgery were 200 minutes and 180 minutes, respectively, while the maximum durations were 237 minutes for anesthesia and 209 minutes for surgery.

Postoperative 6th- and 24th-hour PONV scores, Visual Analog Scale (VAS) nausea scores, and VAS sore throat scores for all patients are presented in Tables 3 and 4.

**Table 3:** PONV and VAS assessments of male and female patients

	Male (M) (*n*=54)			Female (F) (*n*=98)			Effect Size
	Mean±SD	95% CI (min-max)	Median (min-max)	Mean±SD	95% CI (min-max)	Median (min-max)	Cohen’s d (M/F)
PONV 6h	1.98±1.67	1.53–2.44	1 (0–6)	2.73±2.00	2.33–3.14	2 (0–6)	−0.4
PONV 24h	1.48±1.34	1.12–1.85	1 (0–5)	1.47±1.50	1.17–1.77	1 (0–6)	0.01
VAS nausea 6h	15.98±23.87	9.47–22.50	1.5 (0–100)	34.51±31.30	28.23–40.79	32.5 (0–100)	−0.64
VAS nausea 24h	11.93±20.40	6.36–17.49	0 (0–70)	17.54±21.18	13.30–21.79	10 (0–80)	−0.27
VAS sore throat 6h	30.30±31.50	21.70–38.90	19 (0–100)	48.31±28.27	42.64–53.97	50 (0–100)	−0.61
VAS sore throat 24h	22.22±24.12	15.64–28.81	11.5 (0–85)	37.97±25.84	32.79–43.15	36 (0–100)	−0.62

**Table 4:** PONV and VAS assessments in patients with and without pharyngeal tampon

	Pharyngeal Tampon (+)			Pharyngeal Tampon (-)			Effect Size
	Mean±SD	95% CI (min-max)	Median (min-max)	Mean±SD	95% CI (min-max)	Median (min–max)	Cohen’s d (+/-)
PONV 6h	2.33±1.82	1.90–2.76	2 (0–6)	2.59±2.01	2.14–3.03	2 (0–6)	−0.14
PONV 24h	1.30±1.40	0.97–1.63	1 (0–6)	1.62±1.47	1.30–1.95	1 (0–6)	−0.23
VAS nausea 6h	25.86±29.27	18.88–32.84	15 (0–100)	29.70±30.93	22.90–36.49	20 (0–100)	−0.13
VAS nausea 24h	14.41±20.67	9.49–9.34	3 (0–80)	16.51±21.38	11.81–21.21	5 (0–70)	−0.1
VAS sore throat 6h	47.25±30.92	39.88–54.63	49.5 (0–100)	37.34±29.75	30.80–43.88	34 (0–100)	0.32
VAS sore throat 24h	34.17±25.10	28.19–40.16	30.5 (0–96)	30.84±27.29	24.84-36.84	29.5 (0-100)	0.13

Patients were additionally categorized into PONV severity categories based on the PONV impact score for clinical interpretation: mild/no PONV (score < 5) and moderate-to-severe PONV (score ≥ 5). These severity categories are distinct from randomized allocation (pharyngeal tampon group vs no tampon group). At 6 hours postoperatively, 120 patients were classified as mild/no PONV and 32 as moderate-to-severe PONV. By 24 hours, 146 patients were classified as mild/no PONV and 6 as moderate-to-severe PONV.

When outcomes were compared between the pharyngeal tampon and no tampon groups, there were no statistically significant differences in nausea–vomiting at either time point (6th hour: *p* = 0.76; 24th hour: *p* = 0.68). Likewise, PONV scores at 6 and 24 postoperative hours did not differ significantly by tampon placement (*p* = 0.66 and *p* = 0.12, respectively).

VAS nausea-vomiting scores were also compared between the pharyngeal tampon and no tampon groups. There were no statistically significant differences at either time point (6th hour: *p* = 0.46; 24th hour: *p* = 0.55)

When sore throat was compared between the pharyngeal tampon and no tampon groups, VAS sore throat scores were higher in the pharyngeal tampon group at both time points; however, this difference was not statistically significant at the 6th and 24th hour (*p*=0.04—not significant after Bonferroni correction (*α*=0.025)—and *p*=0.35). Despite loss of statistical significance after Bonferroni correction, the mean difference in 6-hour sore throat VAS scores was 9.9 points (Cohen’s d = 0.32), indicating a small-to-moderate clinically relevant effect.

When VAS nausea–vomiting scores were compared by sex between 6th-hour and 24th-hour, significantly higher nausea and vomiting scores were observed in female patients (*p*<0.001 and *p*=0.02, respectively).

When examining the effect of female gender on nausea and vomiting, VAS scores for nausea and vomiting were found to be higher in female patients compared to males among those who received throat pack placement, at both the 6th and 24th postoperative hours(p=0.01 and *p*=0.04). Accordingly, even within patients who received throat packing, female sex was associated with higher nausea–vomiting symptom severity, particularly in the early postoperative period. Even when the analysis was restricted to patients who received throat packs, female patients exhibited significantly higher PONV scores compared with male patients, particularly at the 6th postoperative hour (*p*<0.001).

When the 6th-hour and 24th-hour sore throat VAS scores were compared by gender, female patients had statistically significantly higher scores (*p*<0.001).

When examining the effect of gender on postoperative sore throat, VAS sore throat scores were found to be statistically significantly higher in female patients among those who did not receive throat packs, at both the 6th and 24th postoperative hours (*p*<0.001 and *p*<0.001). Even when analyzing only patients who did not receive throat pack placement, female patients reported more frequent sore throat complaints compared to male patients.

No significant correlation was found between age and PONV scores, or VAS scores for nausea, vomiting, and sore throat (*p*>0.05).

## Discussion

PONV is a significant complaint among septorhinoplasty patients, as it can delay hospital discharge and prolong the return to daily routines. As septorhinoplasty is a satisfaction-oriented procedure, the absence of PONV and the rapid return of patients to daily life are important goals in the postoperative period.

Numerous studies have investigated the effects of throat packing on PONV during nasal surgeries [11–14]. Unfortunately, few studies have investigated the effects of throat packing on PONV within a single subtype of nasal surgery. Our study is valuable in that it specifically evaluates throat packing and postoperative sore throat in septorhinoplasty patients, while also highlighting gender as a contributing factor. To our knowledge, no prior study has addressed the impact of gender on these outcomes in septorhinoplasty, making this work an important contribution to an underexplored area.

In our study, we found that throat pack placement in septorhinoplasty patients did not reduce PONV and was associated with an increase in postoperative sore throat complaints. Because all patients received contemporary dual prophylaxis, the absence of an intergroup difference should be interpreted as indicating no additional benefit of throat packing under standard antiemetic care, while the effect of antiemetic medications cannot be fully excluded. Similar findings were reported in a recent study by Borna et al., which also investigated septorhinoplasty patients [3]. In the study conducted by Meco et al., it was concluded that throat pack placement during sinonasal surgeries had no significant effect on either PONV or 24th-hour postoperative throat pain [12]. In our study, throat pain was found to be higher in patients who received throat packs, at both the 6th and 24th postoperative hour, but this was not statistically significant.

Comprehensive meta-analyses and systematic reviews have reported that throat pack placement has no significant effect on PONV and may increase postoperative throat pain, which is consistent with the findings of our study [2, 15]. In light of all these findings, we believe that throat pack placement in septorhinoplasty patients is an unnecessary practice, as it does not reduce PONV and is associated with an increase in postoperative throat pain. Although throat packing may help prevent blood swallowing, we suggest that it does not alleviate PONV, possibly because irritation of the pharyngeal region may in fact exacerbate the nausea and vomiting reflex [13]. Our study demonstrates that the use of a throat pack does not reduce PONV but may increase postoperative throat pain, and confirms these findings in septorhinoplasty patients with results consistent with and supportive of previous studies. These findings should be interpreted within the early postoperative window (6–24 hrs), as delayed PONV and longer-term throat discomfort were not assessed.

Another important points are that mucosal trauma associated with endotracheal intubation is a well-recognized contributor to postoperative sore throat [8]. Supporting this, studies of rhinoplasty performed under conscious sedation—where airway instrumentation is avoided—report that postoperative sore throat is exceedingly rare, highlighting the central role of the endotracheal tube in pharyngeal discomfort [16–18]. Although the use of a laryngeal mask airway (LMA) may theoretically reduce postoperative throat symptoms by minimizing mucosal irritation, its application in septorhinoplasty remains limited because LMAs do not provide adequate airway protection in procedures with significant bleeding potential [3, 11]. In light of these considerations, meticulous intubation, minimization of mucosal trauma, the use of appropriately sized endotracheal tubes, and careful cuff-pressure management may reduce postoperative sore throat in rhinoplasties performed under general anesthesia [8].

Current studies indicate that nausea and vomiting are more commonly observed in female patients following various surgical procedures [6, 19, 20]. Although findings in the literature remain controversial, factors such as sex hormone levels (particularly estrogen), preoperative psychosocial status, and pharmacogenomic gene polymorphisms are considered the most significant parameters influencing the incidence of PONV in women [6]. The higher incidence of PONV in female patients has been attributed to the effects of estrogen and progesterone on serotonergic and dopaminergic pathways in the central nervous system, which lower the threshold for nausea and vomiting, as well as to sex-related differences in the pharmacokinetics of antiemetic agents and psychosocial factors [6, 7]. There is also evidence that hormonal fluctuations in women can influence gastroesophageal motility and gastric rhythm, potentially increasing susceptibility to nausea and vomiting [21]. Clinical studies evaluating the association between menstrual cycle phase and PONV have reported conflicting results; notably, a recent prospective cohort study using progesterone-confirmed cycle phase found no significant differences in postoperative nausea and vomiting between luteal and non-luteal phases [22]. Although menstrual cycle phase and hormonal status were not assessed in our study, such variability would be expected to increase within-group heterogeneity among female participants and potentially attenuate detectable between-sex differences. Therefore, the observed sex-related differences remain clinically informative, while future studies incorporating hormonal profiling are needed to clarify underlying mechanisms.

From a pharmacokinetic (PK) perspective, sex-related differences in body composition and fat distribution, hepatic metabolism, and drug transport processes may alter the absorption, distribution, metabolism, and elimination of anesthetics, antiemetics, and other perioperative medications, thereby influencing effective drug exposure and duration of action [7]. From a pharmacodynamic (PD) perspective, sex-related differences in the sensitivity of emesis-related receptors and central neurotransmitter systems—including serotonergic and dopaminergic pathways—may modify the emetogenic threshold and influence clinical responses to perioperative drugs and prophylactic antiemetic regimens [7]. Collectively, these PK/PD factors provide a biologically plausible framework for why female patients may experience a higher PONV burden even under standardized prophylaxis, although dedicated mechanistic studies are needed to confirm causal pathways.

In addition, it is widely recognized that women’s narrower tracheal anatomy, the mucosal permeability-enhancing effects of estrogen, differences in pain perception, and psychological factors contribute to the increased incidence of postoperative sore throat [8, 9].

Recent studies have primarily focused on gene polymorphisms as a potential explanation for the increased prevalence of PONV in female patients; however, it is evident that further, more targeted research is required to clarify this relationship [19, 23].

Unlike most previous studies, which primarily focused on the effects of throat packing on postoperative nausea, vomiting, and sore throat in nasal surgeries, our study highlights the role of gender as a significant factor in septorhinoplasty patients. We found that female patients consistently reported higher rates of both PONV and postoperative throat pain, regardless of throat pack placement.

To the best of our knowledge, this is the first randomized controlled trial to emphasize the influence of gender on these postoperative outcomes specifically in septorhinoplasty, a procedure in which patient comfort and satisfaction are particularly important. This distinctive finding not only underscores the need to consider gender-specific risk profiles in perioperative care but also adds novel evidence to the existing literature.

In addition, female patients consistently demonstrated higher VAS nausea and sore throat scores compared to male patients, with effect sizes ranging from moderate to large. These findings suggest that gender is not only a statistically significant factor but also a clinically relevant determinant of postoperative comfort. Importantly, the moderate-to-large effect sizes observed in this study reinforce the clinical importance of these gender-related differences, underscoring the need for personalized perioperative management strategies.

In light of these findings, we believe that implementing tailored antiemetic strategies—such as multimodal prophylaxis and consideration of higher-dose or combination therapy—in female patients, together with adopting anesthetic techniques that minimize mucosal trauma (including optimization of endotracheal tube size, careful cuff-pressure monitoring, reduced pharyngeal manipulation, and, when feasible, the use of less traumatic airway devices), may enhance postoperative comfort and ultimately improve overall satisfaction following septorhinoplasty.

Furthermore, tailoring postoperative antiemetic and analgesic strategies for female patients—particularly in septorhinoplasty, a satisfaction-oriented procedure—based on these findings may help reduce postoperative hospital stay and facilitate a faster return to daily life. Although the female gender factor clearly emerged in our study, further hormonal, psychosocial, and pharmacogenomic research is needed to elucidate the underlying causes and provide more objective data.

In our study, no significant correlation was found between age and the incidence of PONV or sore throat. Although there are conflicting views in the literature regarding the relationship between age and PONV, our findings are consistent with those reported by Elsaid et al. [24].

We believe that the absence of elderly patients in our study group is a limiting factor in drawing objective conclusions on this issue. Additionally, there are studies in the literature suggesting that PONV occurs less frequently in elderly patients compared to younger individuals [7, 20]. We believe that more comprehensive studies involving older patients are needed to better understand the effects of age on PONV in septorhinoplasty patients.

While the overall conclusion regarding throat packing and PONV is consistent with prior reports, the key contribution of the present study is its explicit sex-stratified perspective in a septorhinoplasty population. To our knowledge, this is among the first randomized evaluations in septorhinoplasty to report PONV and postoperative throat discomfort outcomes with a dedicated focus on sex-based differences. These findings provide clinically relevant information for perioperative counseling and risk-aware management in an aesthetic surgery setting.

## Limitations

This study has several limitations that should be acknowledged. First, its single-center design may limit the generalizability of the findings to other institutions or patient populations. Anesthesiologist blinding was not feasible, which may introduce performance bias. Although intraoperative care was protocolized to minimize discretionary differences, this source of bias cannot be fully excluded.

Second, outcomes were assessed only at 6 and 24 hours postoperatively; therefore, we cannot draw conclusions about delayed PONV or the longer-term course of postoperative sore throat. Future studies incorporating extended follow-up (e.g., 48–72 hours and later) are needed to better characterize symptom trajectories.

Finally, universal administration of dexamethasone and ondansetron may have attenuated between-group differences in PONV and limited our ability to detect a small incremental effect of throat packing (i.e., a potential type II error). Nevertheless, standardizing prophylaxis enhances the clinical applicability of our findings by reflecting current routine practice.

Future multicenter studies with longer follow-up periods, more inclusive eligibility criteria, and variable antiemetic protocols are warranted to confirm and expand upon these findings.

## Conclusion

In our study, consistent with previous research, throat packing was found to have no significant effect on reducing PONV and was associated with an increased incidence of postoperative throat pain. Therefore, throat packing is not routinely indicated in elective septorhinoplasty.

Moreover, based on our findings, PONV and postoperative throat pain were more frequently observed in female patients. Accordingly, we recommend that postoperative analgesic and antiemetic regimens be tailored to patient gender, with particular consideration given to optimizing symptom management in women.
